# Potential therapeutic effects of *N*-butylidenephthalide from *Radix Angelica Sinensis* (*Danggui*) in human bladder cancer cells

**DOI:** 10.1186/s12906-017-2034-3

**Published:** 2017-12-06

**Authors:** Sheng-Chun Chiu, Tsung-Lang Chiu, Sung-Ying Huang, Shu-Fang Chang, Shee-Ping Chen, Cheng-Yoong Pang, Teng-Fu Hsieh

**Affiliations:** 1 Department of Research, Taichung Tzu Chi Hospital, Buddhist Tzu Chi Medical Foundation, Taichung, Taiwan; 2 Department of Laboratory Medicine, Taichung Tzu Chi Hospital, Buddhist Tzu Chi Medical Foundation, Taichung, Taiwan; 3 General Education Center, Tzu Chi University of Science and Technology, Hualien, Taiwan; 4 Division of Neuro-Oncology, Neuro-Medical Scientific Center, Hualien Tzu Chi Hospital, Buddhist Tzu Chi Medical Foundation, Hualien, Taiwan; 5 Department of Ophthalmology, Mackay Memorial Hospital, Hsinchu, Taiwan; 6 Tzu Chi Stem Cells Center, Hualien Tzu Chi Hospital, Buddhist Tzu Chi Medical Foundation, Hualien, Taiwan; 7 Department of Medical Research, Hualien Tzu Chi Hospital, Buddhist Tzu Chi Medical Foundation, Hualien, Taiwan; 8 Institute of Medical Sciences, School of Medicine, Tzu Chi University, 701, Section 3, Chung-Yang Road, Hualien, 970 Taiwan; 9 Department of Urology, Taichung Tzu Chi Hospital, Buddhist Tzu Chi Medical Foundation, Section 1, Fengxing Road, Tanzi Dist., Taichung City, 427 Taiwan; 10 School of Medicine, Tzu Chi University, Hualien, Taiwan

**Keywords:** Apoptosis, Bladder cancer, Combination therapy, Metastasis, *n*-butylidenephthalide, NHIRD

## Abstract

**Background:**

N-butylidenephthalide (BP) isolated from *Radix Angelica Sinensis* (Danggui) exhibits anti-tumorigenic effect in various cancer cells both in vivo and in vitro. The effect of BP in bladder cancer treatment is still unclear and worth for further investigate.

**Methods:**

Changes of patients with bladder cancer after *Angelica Sinensis* exposure were evaluated by analysis of Taiwan’s National Health Insurance Research Database (NHIRD) database. The anti-proliferative effect of BP on human bladder cancer cells was investigated and their cell cycle profiles after BP treatment were determined by flow cytometry. BP-induced apoptosis was demonstrated by Annexin V-FITC staining and TUNEL assay, while the expressions of apoptosis-related proteins were determined by western blot. The migration inhibitory effect of BP on human bladder cancer cells were shown by trans-well and wound healing assays. Tumor model in NOD-SCID mice were induced by injection of BFTC human bladder cancer cells.

**Results:**

The correlation of taking *Angelica sinensis* and the incidence of bladder cancer in NHIRD imply that this herbal product is worth for further investigation. BP caused bladder cancer cell death in a time- and dose- dependent manner and induced apoptosis via the activation of caspase-9 and caspase-3. BP also suppressed the migration of bladder cancer cells as revealed by the trans-well and wound healing assays. Up-regulation of E-cadherin and down-regulation of N-cadherin were evidenced by real-time RT-PCR analysis after BP treatment in vitro. Besides, in combination with BP, the sensitivity of these bladder cancer cells to cisplatin increased significantly. BP also suppressed BFTC xenograft tumor growth, and caused 44.2% reduction of tumor volume after treatment for 26 days.

**Conclusions:**

BP caused bladder cancer cell death through activation of mitochondria-intrinsic pathway. BP also suppressed the migration and invasion of these cells, probably by modulating EMT-related genes. Furthermore, combination therapy of BP with a lower dose of cisplatin significantly inhibited the growth of these bladder cancer cell lines. The incidence of bladder cancer decreased in patients who were exposed to *Angelica sinensis*, suggesting that BP could serve as a potential adjuvant in bladder cancer therapy regimen.

**Electronic supplementary material:**

The online version of this article (10.1186/s12906-017-2034-3) contains supplementary material, which is available to authorized users.

## Background

Bladder cancer is one of the common cancer in the United States and many other parts of the world [1, 2]. The estimated numbers of new cases are expected to reach 74,000/year in 2015 in the United States [2]. Previous studies showed that approximately 30% of newly diagnosed superficial bladder cancers were multifocal, however, 60–70% of these superficial bladder cancers recurred, and 10–20% of them would undergo stage progression to a muscle-invasive or metastatic disease [3]. The effect of chemotherapy or other systemic treatment for bladder cancer is limited [4]. Although Platinum-based chemotherapy is commonly used for bladder cancer [5, 6], radical cystectomy and systemic chemotherapy are suggested for invasive bladder cancer. These treatment regimens usually fail in 95% patients: at least half of the invasive bladder cancer patients still die of metastases within 2 years after diagnosis, and with less than 10% 5-year survival rate [2, 7].

Several nutrients and non-nutritive phytochemicals have been evaluated in interventional trials for their potential as cancer chemo-preventive agents. Non-traditional treatments using herbs and dietary supplements have also been considered as alternative therapy for cancer. *Angelica sinensis* (Oliv.) Diels (Umbelliferae), which is pronounced as “Danggui” in Mandarin, is one of the most commonly used herbs in traditional Chinese medicine (TCM). It is clinically administrated to replenish blood and to treat several gynecological symptoms such as menstrual disorders in women. *N*-butylidenephthalide (BP), an active compound isolated from the chloroform extract of *Angelica sinensis*, has been identified as having growth-inhibitory and apoptosis-induction effects on various cancer cells [8–13]*.* These findings highlight the potential therapeutic role of BP in clinical application. However, the effect of BP on human bladder cancer cells is still unclear and worth further investigation.

Our aim in this study was to investigate the probable anti-proliferative effect of BP on bladder cancer cells, and to determine the signaling pathway that might involve. In addition, NOD-SCID mice xenograft tumor model was used to evaluate the antitumor effect of BP on bladder cancer in vivo. On the other hand, since Taiwan’s National Health Insurance Research Database (NHIRD) has been successfully used in epidemiological studies of cancer and Chinese herbal products (CHPs) [14, 15], we also investigated the correlation of taking *Angelica sinensis* and the incidence of bladder cancer in Taiwan.

## Methods

Cell proliferation assay, western blot and cell cycle analysis were performed as previously described [9], with further details were provided in Additional file 1. For RNA isolation, cell migration and invasion assay, and quantitative RT-PCR, see Additional file 1.

### Cell culture

Human bladder cancer cell line TCCSUP was purchased from ATCC (American Type Culture Collection, Manassas, VA). Human bladder cancer cell lines 5637, T24, and BFTC (BFTC 905) were purchased from BCRC (Bioresource Collection and Research Center, Hsinchu, Taiwan). Cells were cultured in appropriate culture medium and supplements according to the suggestion of ATCC and BCRC, respectively. Cell lines were authenticated annually by short-tandem repeat analysis and routinely tested for mycoplasma contamination (BCRC).

### Chemicals and antibodies

BP (C_12_H_12_O_2_, 95%) was purchased from Lancaster Synthesis Ltd. (Newgate Morecambe, UK). Cisplatin, dimethyl sulfoxide (DMSO), [3-(4,5-dimethyl thizol-2-yl)-2,5-diphenyl tetrazolium bromide] (MTT), crystal violet, DSD, Tween-20, methanol, and horseradish peroxidase-conjugated secondary antibodies were purchased from Sigma Chemical Co. (St. Louis, MO, USA). The primary antibodies were all purchased from Cell Signaling Technology, Inc., (Danvers, MA, USA). Polyvinyldifluoride (PVDF) membranes, BSA protein assay kit and chemiluminescence reagents were purchased from Amersham Biosciences (Arlington Heights, IL, USA).

### TUNEL assay

Human bladder cancer cells were cultured in the presence or absence of BP (60 μg/ml) for 72 h and then examined for apoptosis with TUNEL assay (In Situ Cell Death Detection Kit, Roche) according to the manufacturer’s instructions.

### Annexin V-FITC staining

Human bladder cancer cells were cultured in the presence or absence of BP (60 μg/ml) for 3, 18 and 24 h, as indicated. The vehicle control group was treated with 0.2% DMSO only. Apoptotic cell death was examined using annexin V-FITC detection kits according to the manufacturer’s instructions (BD Biosciences, San Diego, CA, USA). Ten thousand events were acquired for each sample and analyzed by Accuri C6 flow cytometer with CFlow® software.

### Animal studies

Tumors were generated as previously described [9] and further details were provided in Additional file 1.

### Patients and study design

Taiwan implemented a National Health Insurance (NHI) program in 1995 to provide comprehensive health care coverage. Enrollment in this government-run, universal, single-payer insurance system is mandatory and up to 99% of the 23 million residents of Taiwan receive medical care through the NHI program. In addition, >97% of the hospitals and clinics in Taiwan are contracted to provide health care services under the NHI [16]. All data related to these services are collected and input into the NHIRD by the National Health Research Institute to provide a comprehensive record of medical care. The data consist of ambulatory care records, in-patient care records, and registration files of the insured, and the database includes all claims data from the NHI program. The NHI Bureau randomly reviews the charts of one out of every 100 ambulatory cases and one out of every 20 in-patient cases, as well as conduct patient interviews to verify the accuracy of the diagnosis [17].

This study used the NHIRD collected within 2003 to 2009. The study design featured a study cohort and a comparison cohort. Patients with newly-diagnosed with bladder cancer (International Classification of Diseases, Ninth Revision, Clinical Modification (ICD-9-CM) code 188.XX) before the index date were excluded. Patients who received CHPs containing *Angelica sinensis* between 2002 and 2009 were then identified as the study cohort (the exposure group). The date of initiation of *Angelica sinensis* exposure was used as the patient’s index date. The control cohort (the non-exposure group) included all of the other patients without taking CHPs therapy, and propensity score matching with age and gender was applied to select the controls.

### Charlson Comorbidity Index Score

The Charlson Comorbidity Index Score (CCIS) is a widely accepted measure for risk adjustment in administrative claims data sets [18–20]. The CCIS were calculated for each patient by assigning 1 point each for myocardial infarct, congestive heart failure, peripheral vascular disease, dementia, cerebrovascular disease, chronic lung disease, connective tissue disease, ulcer, chronic liver disease and diabetes by assigning 2 points each for hemiplegia, moderate or severe kidney disease, diabetes with end organ damage, tumor, leukemia and lymphoma, 3 points for moderate or severe liver disease and 6 points each for malignant tumor, metastasis and acquired immune deficiency syndrome.

### Other variables

The study subjects were classified into three groups: (1) low SES, < 583 US $ per month (New Taiwan Dollars [NTD] ~17,500); (2) moderate SES, 583–833 US $ per month (NTD 17500–25,000); and (3) high SES, ≥ 833 US $ per month (≥ NTD 25001) [21]. Low income was set at NTD 17500 because this was the government-stipulated minimum wage for full-time employees in Taiwan in 2009. The geographic regions where the individuals resided were recorded as northern, central, southern, and eastern Taiwan.

### Statistical analysis

All data were shown as mean ± S.D. Statistical differences were analyzed using the Student’s t-test for normally distributed values and by nonparametric Mann–Whitney *U*-test for values with a non-normal distribution. Significant differences between groups were evaluated using analysis of variance (ANOVA) with Games-Howell test as *post-hoc* test. Pearson’s chi-square test was used for categorical variables such as gender, SES, geographic region of residence, co-morbidities and incidence of bladder cancer.

## Results

### The incidence of bladder cancer with or without *Angelica sinensis* exposure in NHIRD database

From 2002 to 2009, NHIRD data from 17,091 patients who had taken *Angelica sinensis* (solely or as one of the ingredients) were included for analysis. Table 1 shows the number of patients, age, gender, and distribution of patients in different comorbidities. The mean age at diagnosis was 53.8 ± 10.9 years. Severe comorbidity (CCIS ≧ 4) was noted in 4.5% patients who were exposed to *Angelica sinensis*. At the end of the follow-up period, 30 patients had bladder cancer, including 8 (0.05%) in the exposure group, and 22 (0.13%) in the control group (Table 2; *p* = 0.0106, Relative risk = 0.38). The result prompted us to investigate the physiological effect of BP, one of the major component found in the chloroform extract fraction of *Angelica sinensis*, on human bladder cancer cells.

**Table 1:** Characteristics of the patients with or without *Angelica sinensis* exposure from 2002 to 2009 in Taiwan (*n* = 34,182)

Variables	Non-exposure N (%)	Exposure N (%)	*p*-value
Total	17,091 (50.0)	17,091 (50.0)	
Mean age,years(±SD)	53.8 ± 10.9	53.8 ± 10.9	1.00
Sex			1.00
Male	6244 (36.5)	6244 (36.5)	
Female	10,847 (63.5)	10,847 (63.5)	
CCIS score			<.0001
Mean ± SD	0.40 ± 1.00	0.76 ± 1.43	
0–1	15,523 (90.8)	14,174 (82.9)	
2–3	1259 (7.4)	5157 (12.6)	
Over 4	309 (1.8)	760 (4.5)	
Comborbidities			
Diabetes mellitus	1181 (6.9)	1590 (9.3)	<.0001
Socioeconomic status			<.0001
Low SES(<=17,500)	7510 (43.9)	6954 (40.6)	
ModerateSES (17,500~25,000)	5979 (35.0)	6249 (36.6)	
High SES(> = 25,000)	3602 (21.1)	3897 (22.8)	
Urbanization			<.0001
Urban	5428 (31.8)	5130 (30.0)	
Suburban	7390 (43.2)	7872 (46.1)	
Rural	4273 (25.0)	4089 (23.9)	
Geographic region			<.0001
Northern	9090 (53.2)	6672 (39.0)	
Central	2204 (12.9)	4728 (27.7)	
Southern	5335 (31.2)	5319 (31.1)	
Eastern	462 (2.7)	372 (2.2)

**Table 2:** Bladder cancer incidence and relative risk

Characteristics	Bladder cancer N (%)	Relative risk	*p*-value
Non-exposure to *Angelica sinensis*	22 (0.13%)	1	0.01
Exposure to *Angelica sinensis*	8 (0.05%)	0.38	

### BP inhibited proliferation of human bladder cancer cells

**Fig. 1:**
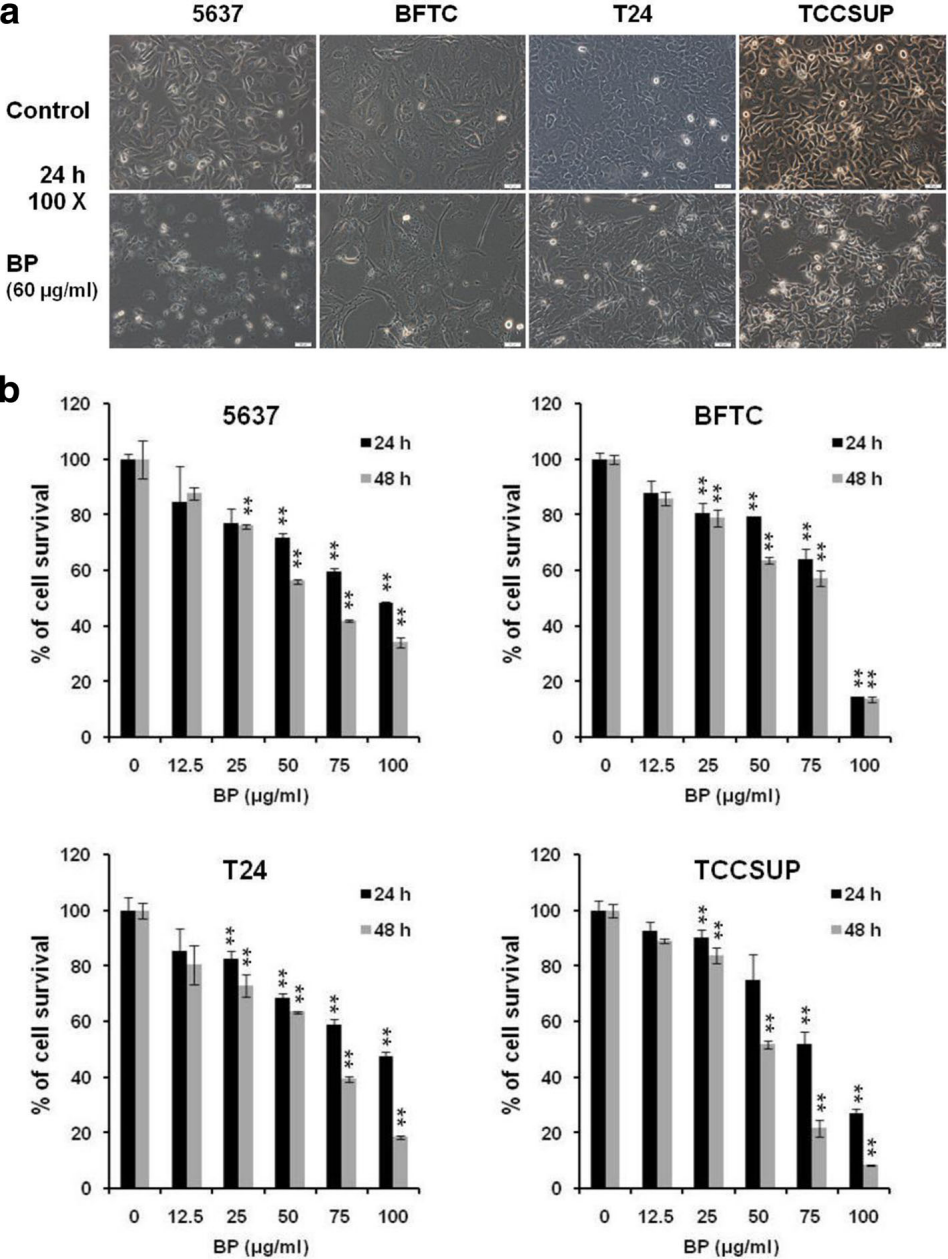
Effects of BP on the viability of human bladder cancer cells. **a** Human bladder cancer cells (5637, BFTC, T24 and TCCSUP) were treated with 0.2% DMSO as vehicle control or 60 μg/ml BP for 24 h, were shown; Scale bar: 50 μm. **b** Human bladder cancer cells were treated with various concentration of BP (12.5 to 100 μg/ml) for 24 (■) and 48 h (■), respectively, and the survival rate was evaluated with MTT assay. Data are presented as means ± S.D. obtained from three different experiments. ** *p* < 0.01 versus vehicle

To determine the cytotoxicity effect of BP on bladder cancer cells, 4 human bladder cancer cell lines (5637, BFTC, T24, and TCCSUP) were treated with increasing concentration (12.5 to 100 μg/ml) of BP for 24 and 48 h, respectively, and subsequently evaluated for cell viability using the MTT assay. As shown in Fig. 1a, all 4 bladder cancer cell lines showed rounding up and shrinkage of cells as compared to the control cells after 24 h of BP treatment. We further demonstrated that BP significantly reduced the viability of 4 bladder cancer cell lines in a dose- and time-dependent manner by MTT assay (Fig. 1b). Treatment of 4 bladder cancer cells with 50 or 75 μg/ml BP for 48 h resulted in 56.3% and 42% (5637), 63.7% and 57.4% (BFTC), 63.5% and 39.4% (T24), 51.9% and 21.6% (TCCSUP) cell survival, respectively. The IC_50_ at 48 h of BP treatment in bladder cancer cells were: 5637, 67.1 μg/ml; BFTC, 64.1 μg/ml; T24, 59.9 μg/ml; TCCSUP, 53 μg/ml, respectively. Thus, we used 60 μg/ml BP in the subsequent experiments.

### BP induced sub-G1 accumulation in human bladder cancer cells

**Fig. 2:**
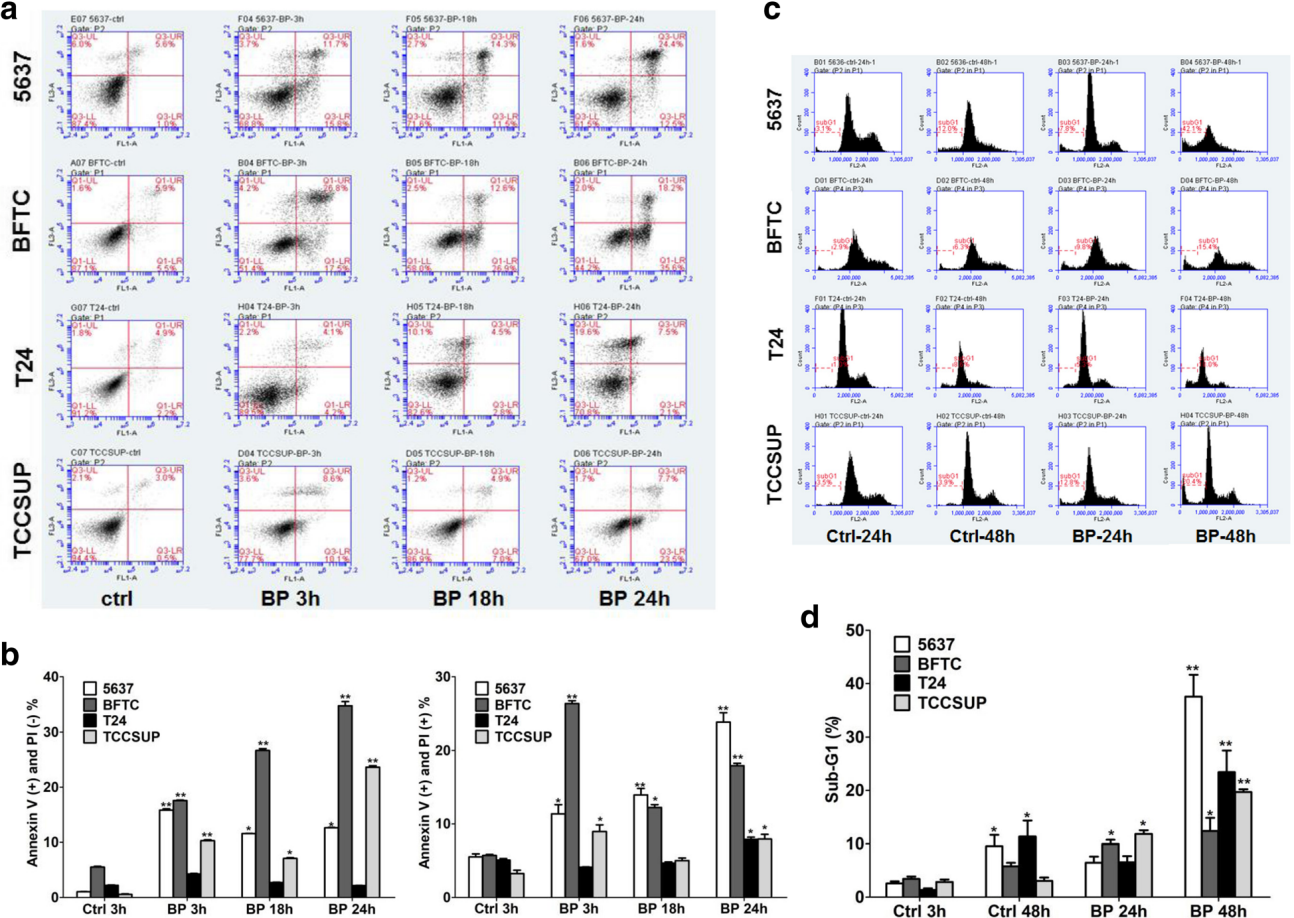
Flow cytometric analysis of bladder cancer cells treated with BP. **a** Human bladder cancer cells were analyzed by annexin V-FITC staining in the vehicle control group (0.2% DMSO) for 3 h or at presence of 60 μg/ml BP for 3, 18 and 24 h. **b** The percentage of annexin V-FITC positive population in bladder cancer cells in **a** is shown. **c** The accumulation of sub-G1 cell population in the presence or absence of 60 μg/ml BP for 24 and 48 h. **d** The percentage of sub-G1 population in bladder cancer cells in **c** is shown

To investigate the role of apoptosis in BP-induced bladder cancer cell death, annexin V-FITC staining and flow cytometric analysis was performed (Fig. 2). Human bladder cancer cells treated with 60 μg/ml BP for 0 to 24 h were analyzed by flow cytometry (Fig. 2a). The annexin V-FITC positive and PI negative populations (Q3-LR) were increased after BP treatment as compared to the control group. Apoptosis was noted as early at 3 h after BP treatment in 4 bladder cancer cell lines. After BP treatment for 24 h, the Q3-LR portion increased to 12.6% (5637), 34.8% (BFTC), 2.2% (T24) and 23.6% (TCCSUP), respectively (Fig. 2b). The Q3-LR portion of T24 was significantly lower than other bladder cancer cell lines after BP treatment and worth for further clarification. The appearance of Sub-G1 cell population can be evaluated as the degree of apoptotic cell death. As showed in Fig. 2c, treatment with 60 μg/ml BP induced the accumulation of sub-G1 portion in 4 bladder cancer cell lines. After BP treatment for 48 h, the sub-G1 portion increased to 37.6% (5637), 12.4% (BFTC), 23.4% (T24) and 19.7% (TCCSUP), respectively, in 4 bladder cancer cell lines (Fig. 2d). Together, these data suggested that apoptosis induction was involved in BP-induced bladder cancer cell death.

### BP induced mitochondrial-mediated apoptosis in human bladder cancer cells

**Fig. 3:**
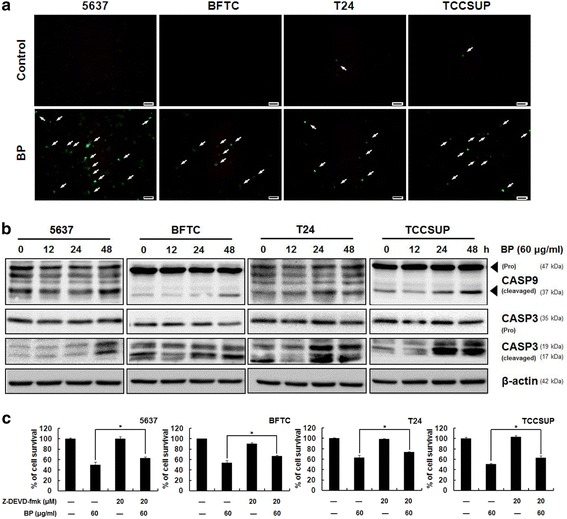
BP induced mitochondrial-mediated apoptosis in human bladder cancer cells. **a** Human bladder cancer cells were treated with 0.2% DMSO (control) or 60 μg/ml BP for 72 h and then stained with the TUNEL assay. TUNEL positive cells (green fluorescence) were indicated by arrows. Scale bar: 50 μm. **b** Human bladder cancer cells were treated with 60 μg/ml BP for 0 to 72 h, and western blot analysis were performed for cleaved caspase-9 and -3. β-actin was used as an internal control. **c** MTT assay of human bladder cancer cells pretreated with caspase 3 inhibitor Z-DEVD-fmk (20 μM) for 1 h and then treated in the presence or absence of 60 μg/ml BP for 48 h. The values are the mean ± S.D. from three independent experiments, * *p* < 0.05 versus vehicle

TUNEL assay was performed to detect apoptotic cells that undergo DNA degradation during the late stage of apoptosis. Cells treated with 60 μg/ml BP for 72 h were evaluated by TUNEL assay. The TUNEL positive cells (green fluorescence) were significantly increased after BP treatment as compared to the control (Fig. 3a). Activation of caspase proteins is the crucial steps for apoptosis induction. The key cysteine proteases, caspase −9 and −3, play critical roles in the activation of mitochondria-mediated apoptosis. The involvement of these caspases in BP-induced apoptosis was investigated in these bladder cancer cell lines. Cleavages of caspase −9 and −3 increased in bladder cancer cells from 0 to 48 h after BP treatment (Fig. 3b). These data suggested that BP induced mitochondria-mediated apoptosis in bladder cancer cells. To examine the pivotal role of caspase 3 in BP-induced apoptosis, cells were pretreated with Z-DEVD-fmk (caspase 3 inhibitor, 20 μM) for 1 h, and then treated with 60 μg/ml BP for 48 h (Fig. 3c). The pretreatment of inhibitor partly blocked BP-induced cell death (63% in 5637, 66.3% in BFTC, 73.6% in T24, and 62.5% in TCCSUP, respectively) as compared to the BP alone group (49.9% in 5637, 53.4% in BFTC, 62.6% in T24, and 50.2% in TCCSUP, respectively). Although to a lesser extent, these data further confirmed that caspase-dependent pathway was involved in BP-induced human bladder cancer cell death.

### BP suppressed human bladder cancer cells migration and invasion in vitro

**Fig. 4:**
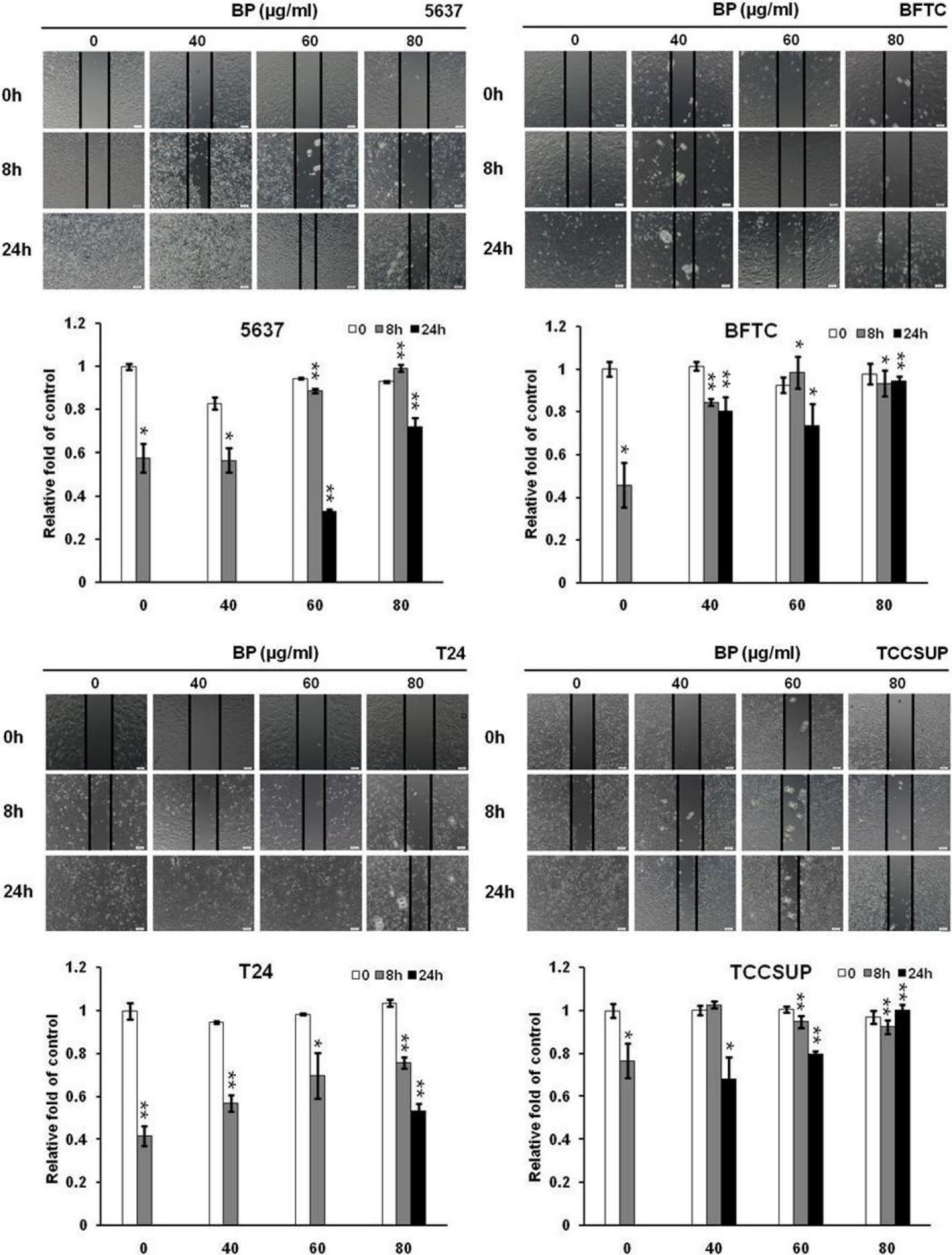
The inhibitory effect of BP on the migration of human bladder cancer cells- wound healing assay. Human bladder cancer cells were treated with 0.2% DMSO as control or 40 to 80 μg/ml BP for the indicated time points. Images of wound closures were captured using inverted microscope with 100 X magnification. The cell-free area invaded by migrated cells across the black lines were calculated by three randomized fields and quantified. The cell-free distance at 0 h were set at as 100%. Data obtained from three independent experiments and presented as mean ± S.D. from three independent experiments. Scale bar: 100 μm, * *p* < 0.05 versus vehicle; ** *p* < 0.01 versus vehicle

The inhibitory effect of BP in bladder cancer cells were further examined by wound closure assay (Fig. 4). The control cells migrated and filled the scratched area within 24 h. The cell-free area filled by migrated cells were quantified and compared. As shown in Fig. 4, these results demonstrated that the migration of BP-treated bladder cancer cells was inhibited: the extents of inhibition of migration by 60 μg/ml BP at 24 h for 5637, BFTC, T24 and TCCSUP were 72%, 94.5%, 53.6% and 100.4%, respectively. These data suggested that BP inhibited the migration of human bladder cancer cells in a time- and dose-dependent manner.

**Fig. 5:**
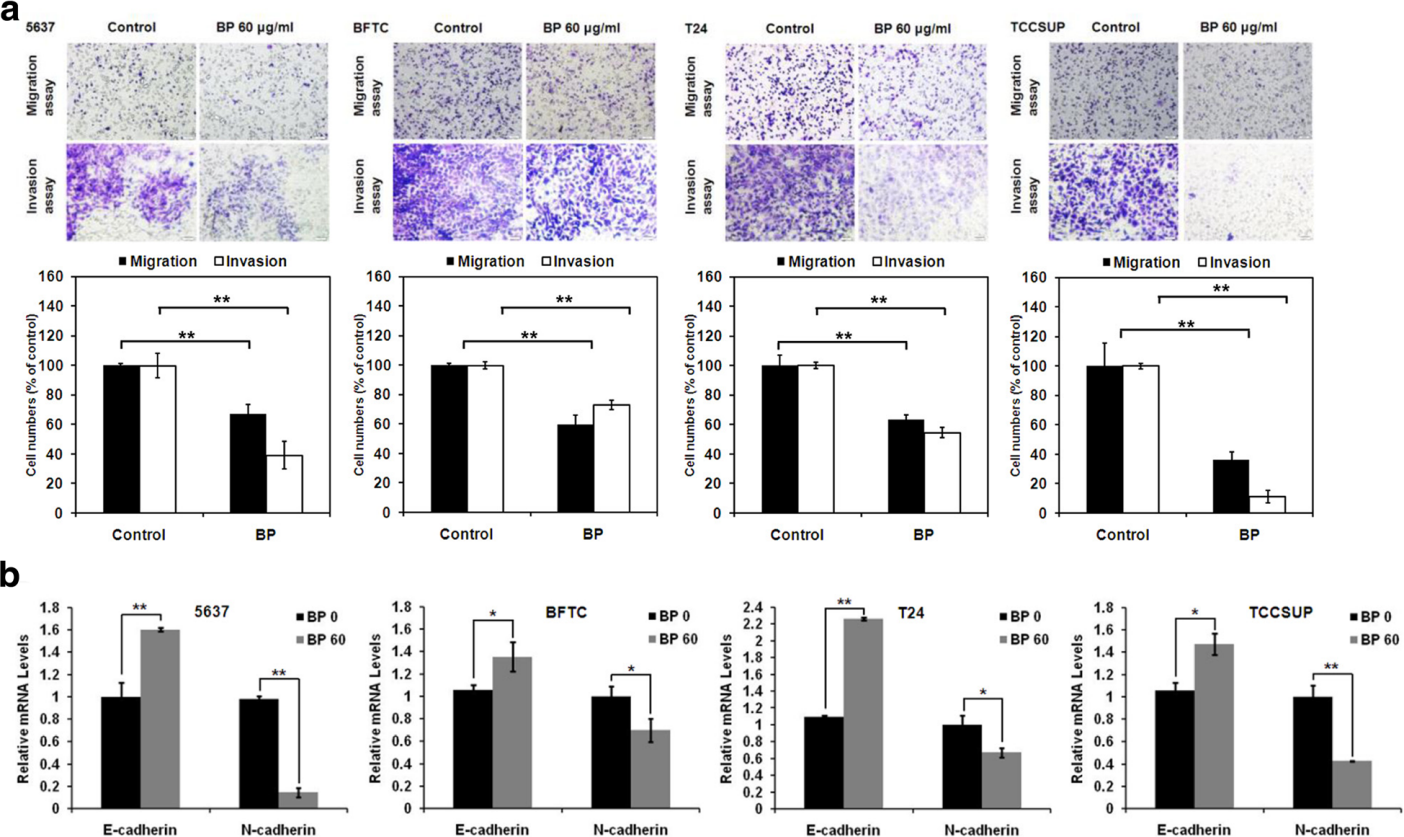
The inhibitory effects of BP on the migration and invasion of human bladder cancer cells- trans-well assay. **a** Human bladder cancer cells were pretreated with 0.2% DMSO as control or 60 μg/ml BP for 24 h and then seeded onto the trans-well hanging insert coated with (invasion) or without (migration) 50 μg/ml Matrigel for different time points (5637: 16 h, BFTC: 2 h, T24: 4 h and TCCSUP: 6 h). Images were were captured using inverted microscope with a 200 X magnification; Scale bar: 50 μm. Quantitative data are shown by histograms of migrated/invaded cells from different treatment. **b** Human bladder cancer cells were treated with 60 μg/ml BP for 24 h, and the mRNA expression levels of E-cadherin and N-cadherin were determined by real-time RT-PCR analysis. Data are presented as means ± S.D. from three different experiments. * *p* < 0.05 versus vehicle; ** *p* < 0.01 versus vehicle

The inhibitory effect of BP on bladder cancer cell was further explored with trans-well migration and invasion assay. The migration of BP-treated (60 μg/ml) human bladder cancer cells decreased significantly as compared to the non-treated cells (Fig. 5a). The migrated cells accounted for 66.9% (5637), 59.6% (BFTC), 63.2% (T24) and 36.1% (TCCSUP), respectively, as compared to the non-treated cells. Similar results were obtained in the invasion assay, the migrated cells accounted for 39% (5637), 72.9% (BFTC), 54.5% (T24) and 11.1% (TCCSUP), respectively, as compared to the control cells. Taken together, we showed that BP suppressed cell migration and invasion of human bladder cancer cells.

### Modulation of epithelial-mesenchymal transition by BP

Modulation of genes that are involved in epithelial-mesenchymal transition (EMT) has been regarded as a hallmark of cancer metastasis. To evaluate whether BP could affect the metastatic potential of bladder cancer cell lines, the expression levels of several EMT-related genes were examined using real-time RT-PCR. Up-regulation of the mRNA expression of epithelial marker E-cadherin after treatment with BP for 24 h were 1.6 fold (5637), 1.35 fold (BFTC), 2.26 fold (T24), and 1.47 fold (TCCSUP), respectively. Down-regulation of the mRNA expression of mesenchymal marker N-cadherin after treatment with BP for 24 h were 0.14 fold (5637), 0.7 fold (BFTC), 0.68 fold (T24), and 0.43 fold (TCCSUP), respectively (Fig. 5b). These results implicated that BP might inhibited the migration in bladder cancer cells via the modulation of EMT genes.

### Effect of BP on the combination therapy with cisplatin

**Fig. 6:**
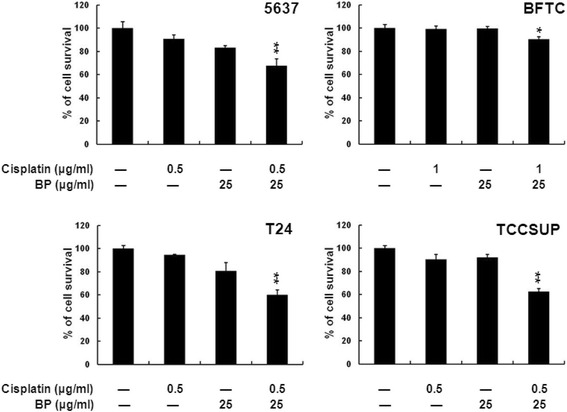
The synergic effects of BP on combination therapy with cisplatin. Human bladder cancer cells were treated with cisplatin or BP only, or a combination therapy for 24 h, and analyzed with MTT assay. Data are presented as means ± S.D. from three different experiments. * *p* < 0.05 versus vehicle; ** *p* < 0.01 versus vehicle

To investigate whether BP could enhance the therapeutic effects of chemotherapy agent such as cisplatin in combination therapy, the low dose of BP and cisplatin were used to evaluate their synergistic efficacy in bladder cancer cells. Human bladder cancer cells treated with cisplatin, or BP, or in combination for 24 h, were analyzed for viability with MTT assay. BP, at a lower dosage (25 μg/ml) showed synergistic cytotoxic effect when combined with cisplatin: 5637: 67.5% (cisplatin alone: 90.5%, BP alone: 83.1%), BFTC: 90.1% (cisplatin alone: 99%, BP alone: 99.3%), T24: 59.8% (cisplatin alone: 94.5%, BP alone: 80.4%) and TCCSUP: 62.4% (cisplatin alone: 90.1%, BP alone: 92%), respectively (Fig. 6). These data implicated the potential role of BP in combination therapy.

### BP suppressed tumor growth in NOD-SCID xenograft mice

**Fig. 7:**
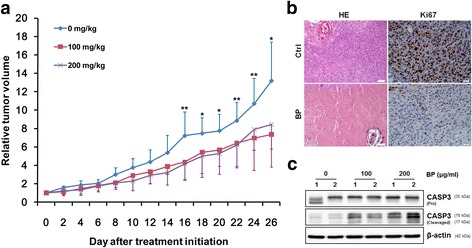
BP inhibits xenographic growth of BFTC cells in NOD-SCID mice. **a** Approximately 5 × 10^5^ BFTC cells were injected into the dorsal subcutaneous tissue of NOD-SCID mice. When the tumor reached 100–250 mm^3^, BFTC tumor-bearing mice were administrated s.c. with vehicle control or 100 to 200 mg/kg BP on days 0–4 for 5 days. The relative tumor volumes of the control and the BP-treated groups are shown as means ± S.D. of tumor volume at each time point. **b** Tumor tissue sections with HE staining. The proliferation marker ki67 were immuno-histochemically identified in the control and the BP-treated groups. Expression of cleavaged caspase-3 in the BFTC xenograft tumor tissue was up-regulated after BP administration as compared to control group by western blotting analysis **c**

To evaluate the antitumor activity of BP in vivo, human bladder cancer xenograft were established by subcutaneous injection of 5 × 10^5^ BFTC cells into the dorsal subcutaneous tissue of NOD-SCID mice. As shown in Fig. 7a, the relative tumor volume in mice treated with 100 and 200 mg/kg BP was smaller than the vehicle treated control mice on day 26 (44.2%, *P* = 0.01; 35.9%, *P* = 0.04, respectively). Hematoxylin/eosin (H&E) staining revealed that control group tumor specimens had large and irregular nuclei and exhibit squamous differentiation which are associated with a poor prognosis as compared to the BP-treated group (Fig. 7b). Furthermore, immunohistochemistry of the proliferation marker, Ki67, demonstrated that the control group showed increased Ki67 expression as compared to the BP-treated group (Fig. 7b). Caspase-3 activation was also observed in BP-treated tumors (Fig. 7c). There were no significant differences of body weight between the control and BP-treated groups.

## Discussion

NHIRD is a population-based dataset which provides researchers to trace the medical service history covering over 99% population in Taiwan, and is widely recognized to provide highly accurate diagnoses and clinical information [22, 23]. Several reports have used the NHIRD to evaluate the associations between different diseases [14, 19, 20]. As there were considerable differences in age, gender and comorbidities between the patients who has or has not been exposed to *Angelica sinensis*, propensity score matching with age and gender was applied to select the controls. The incidence of bladder cancer decreased in the patients who were exposure to *Angelica sinensis* using a matching method. However, the limitations of this study data from NHIRD should, however, be noted. One obvious limitation study is that, the incidence of bladder cancer was low and the numbers of bladder cancer were likely too small for these types of sub-analyses. The other limitations from NHIRD were described in previous studies [19, 20]. Briefly, the severity of bladder cancer cannot be precisely extracted from ICD-9-CM codes and the database does not contain information on tobacco use, dietary habits, carcinogen exposure and body mass index, which may also be risk factors for bladder cancer were the limitations. In addition, the CHPs used might contain additional herbs other than *Angelica Sinensis* and additional bio-active agents. In order to clarify the results from the NHIRD study and the physiological effect of BP on bladder cancer cells, we thus carried out the in vitro and in vivo experiments.

Many dietary phytochemicals and extracts derived from herbs have been tested as antioxidants, or as inhibitors of cancer cell proliferation in vitro and in vivo [24]. Recent reports have demonstrated that BP is cytotoxic to various cancer cells including glioblastoma multiforme (GBM), hepatocellular carcinoma (HCC) and prostate cancer. However, the effect of BP on human bladder cancer cells has not been addressed. Based on the abovementioned discovery that patients who have been exposed to *Angelica sinensis* have lower incidence of bladder cancer, we thus intended to study the probable physiological effect of BP on bladder cancer using bladder cancer cell lines of various stages.

In the subsequent study, we demonstrated the cytotoxic effect of BP on various human bladder cancer cells. BP induced mitochondria-dependent apoptotic cell death, and inhibited the migration of the 4 bladder cancer cell lines. Interestingly, the 4 bladder cancer cell lines were isolated from different stages of bladder cancer. We thus speculated that BP might have therapeutic potential in treating bladder cancer of different stages. However, testing the therapeutic potential of BP in these stages of bladder cancers needs to be evaluated in different mouse models.

The mitochondria-dependent apoptotic cell death was evidenced by caspase-9 and caspase-3 cleavages in a time-dependent manner after BP treatments in these cell lines. Pretreatment of caspase-3 inhibitor (Z-DEVD-fmk) partly rescued the cells from BP cytotoxicity. The late-stage apoptosis was revealed by the TUNEL assay. The ability of BP in eliciting apoptotic cell death had also been documented in brain, lung and prostate cancer cells in previous studies [9–11, 16].

Cell migration is essential for tumor metastasis, and metastasis is the most common fatal complication of all cancer patients [25]. The migration of human bladder cancer cell lines was suppressed by BP in this study, which implicated the anti-metastasis effect of BP on bladder cancer cells. Several crucial steps such as loss of cellular adhesion, increased the motility and invasiveness, has been found to be associated with epithelial-mesenchymal transition (EMT) [26]. The up-regulation of N-cadherin promotes motility, invasiveness and metastasis in tumor cells, whereas the loss of E-cadherin reduces the E-cadherin-mediated cell-cell adhesion and progress toward malignancy. Thus, these events play critical roles in EMT [26]. Our results showed that BP-pretreatment significantly inhibited the migration and invasion of bladder cancer cells (Fig. 5a). In addition, BP suppressed the expression of N-cadherin but activated the expression of E-cadherin in the 4 bladder cancer cell lines (Fig. 5b). These results demonstrated the anti-metastasis effect of BP on bladder cancer cells. We thus speculated that BP might have therapeutic potential in treatment of bladder cancer of all stages.

Synergistic analysis of different anticancer agents is an important approach to determine the ratio and/or dose of drugs for clinical combination application [27]. In previous studies, combination of cisplatin and other drugs could increase the cytotoxicity of chemotherapy in bladder cancer treatment [28, 29]. In the present study, similar result of combined therapy was observed. Bladder cancer cells treated with BP demonstrated increased sensitivity to cisplatin, indicating that BP could be developed as a potential adjuvant cisplatin-based chemotherapy regimen. The mechanism of this synergistic effect includes drug inactivation, alterations in drug target, processing of drug-induced damage and evasion of apoptosis, which all needs to be clarified in the future [30].

## Conclusion

In summary, the present study demonstrates the potential anti-proliferation and anti-metastatic activity of BP on human bladder cancer cells. BP exerted cytotoxicity by inducing apoptosis in human bladder cancer cells: activations of caspases-9 and caspase-3 were evidenced. The anti-metastatic effect of BP in human bladder cancer cells was shown by wound healing and trans-well assays. We have further demonstrated the tumor suppression potential in BFTC-xenograft animal models. In addition, the synergistic cytotoxic effect of BP in combination with cisplatin were also shown. An NHIRD database analysis showed lower incidence of bladder cancer in patients with exposure to *Angelica sinensis,* indicating the therapeutic potential of BP in clinical application.

## Additional file

Additional file 1: Supplementary Methods. (DOC 48 kb)

## Abbreviations

BP: n-butylidenephthalide
NHIRD: National Health Insurance Research Database
