# Supplementary material for: Potential therapeutic effects of N-butylidenephthalide from Radix Angelica Sinensis (Danggui) in human bladder cancer cells
Source: BMC Complement Altern Med. 2017 Dec 6;17:523. doi: 10.1186/s12906-017-2034-3 (PMC5718036; doi:10.1186/s12906-017-2034-3)
Supplement: Additional file 1: — Supplementary Methods. (DOC 48 kb) [file 12906_2017_2034_MOESM1_ESM.doc]

**Supplementary Methods**

**Western blot analysis**

5 x 10^5^ cells per 6-cm petri dish were lysed with 200 μl M-PER mammalian protein extraction reagent containing protease inhibitor cocktail (Thermo Scientific, Rockford, IL, USA) and centrifuged at 13,000 x g at 4 °C for 10 min. The protein concentration in the supernatants was quantified using a BSA Protein Assay Kit. Electrophoresis was performed on a NuPAGE® Bis-Tris Electrophoresis System using 20 μg of protein extract for each lane. Resolved proteins were transferred to PVDF membranes, blocked with 5 % skim milk for 1 h at room temperature, and finally probed with the specific primary antibodies at 4 °C overnight. After the PVDF membrane was washed three times with TBS/0.2 % Tween-20 at room temperature, it was incubated with appropriate secondary antibody labeled with horseradish peroxidase (goat anti-mouse or anti-rabbit, 1:10000, Sigma Chemical, St. Louis, MO, USA) for 1 h at room temperature. All resolved proteins bands were detected using Western Lightning^TM^ Chemiluminescence Reagent Plus (Amersham Biosciences, Arlington Heights, IL, USA).

**MTT assay**

The viability of the cells after treatment with various BP dosages was evaluated using MTT assay preformed in triplicate. Briefly, cells (4 x 10^4^/well) were incubated in 24-well plates containing 0.5 ml of serum-containing medium. Cells were allowed to adhere for 18-24 h and were washed with phosphate-buffered saline (PBS). Solutions were always prepared fresh by dissolving 0.1 % DMSO (control) or BP in culture medium before their addition to cells. The BP -containing medium was removed after treatment for 24 or 48 has indicated, the cells were then washed with PBS, replenished with culture medium containing 300 μg/ml MTT, and incubated for 1 h at 37 °C. After the MTT medium was removed, 0.5 ml of DMSO was added to each well. Absorbance at 570 nm was detected by a multi well plate reader Infinite 200 Pro Tecan^TM^ (Tecan, Mannedorf, Switzerland). The absorbance for DMSO-treated cells was considered as 100 %.

**Cell cycle analysis**

The cell cycle was determined by flow cytometry following DNA staining to reveal the total amount of DNA. Approximately 5 × 10^5^ of bladder cancer cells were incubated with 60 μg/ml BP for the indicated time. Cells were harvested with trypsin/EDTA, collected, washed with PBS, fixed with cold 100% ethanol overnight, and then stained with a solution containing 20 μg/ml PI, 0.2 mg/ml RNase A, and 0.1 % Triton X-100 for 30 min in the dark. The cells were then passed through Accuri C6 flow cytometer to measure the DNA content. The data were obtained and analyzed with CFlow® software.

**Cell migration and invasion assay**

Cell migration was determined by the wound healing and the trans-well assays. For wound healing assay, cells were seeded and grown overnight to 90~95 % confluence in 24-well plates (5637: 3 x 10^4^, BFTC: 7 x 10^3^, T24: 1.5 x 10^4^, TCCSUP: 2.5 x 10^4^). Migration was tested in wound-healing assays using culture inserts (ibidi, Martinsried, Germany). Cells were washed with PBS and cultured with medium containing 0 to 80 μg/ml BP. Wound closure was evaluated and photographed at 0, 8 and 24 h with an inverted microscope (Olympus CKX41 fluorescence microscope, Melville, NY, USA).

The trans-well assay was performed using Hanging inserts (Millipore Co., MA, USA) with 8 μm polycarbonate membrane in a 24-well plate. Cells were seeded in 6 well plates and treated without or with 60 μg/ml BP for 24 h. Cells were then detached and seeded (5 x 10^4^) to the upper chamber of the transwell plates. Upper chambers were filled with serum free medium and lower chambers were filled with cultured medium containing 10 % FBS as a chemo-attractant. Incubation was carried out at 37 ℃ for the indicated time points (5637: 16 h, BFTC: 2 h, T24: 4 h, TCCSUP: 6 h). The hanging inserts were washed with PBS, and cells on the upper filter surface were wiped away with a cotton swab. The inserts were subsequently fixed with 10 % formalin for 10 min at room temperature, stained with 0.2 % *w*/*v* crystal violet, washed with PBS, and counted the remaining cells on the opposite site of the filter under a light microscope operating at 200 X magnification. The migration cell numbers of control group was considered as 100 %.

Cell invasion assay were performed using Hanging inserts (Millipore Co., MA, USA) with 8 μm polycarbonate membrane coated with 50 μg/ml Matrigel® solution in a 24-well plate. Cells were seeded in 6 well plates and treated without or with 60 μg/ml BP for 24 h. Cells were then detached and seeded (5 x 10^4^) to the upper chamber of the transwell plates. Upper chambers were filled with serum free medium and lower chambers were filled with cultured medium containing 10 % FBS as a chemo-attractant. Incubation was carried out at 37 ℃ for the indicated time points (5637: 16 h, BFTC: 2 h, T24: 4 h, TCCSUP: 6 h). The hanging inserts were washed with PBS, and cells on the upper filter surface were wiped away with a cotton swab. Cells that invaded the Matrigel and reached the bottom layers of the chamber were subsequently fixed with 10 % formalin for 10 min at room temperature, stained with 0.2 % *w*/*v* crystal violet, washed with PBS, and counted the remaining cells on the opposite site of the filter under a light microscope operating at 200 X magnification. The invasion cell numbers of control group was considered as 100 %.

**Real-time RT-PCR Analysis**

Total RNA was extracted from cell lines using RNeasy Mini Kit® (Qiagen, Valencia, CA, USA) and reverse transcribed at 37°C for 60 min with Omniscript RT Kit® (Qiagen) according to the manufacturer’s instructions. Real‐time RT‐PCR analysis was performed in triplicate in a Step One Plus Real-Time PCR system (Applied Biosystems, Foster City, CA, USA) with Power SYBR® Green PCR Master Mix (Applied Biosystems) in a final volume of 20 μl/reaction. Threshold cycle (C_t_) value of each tested gene was normalized to the C_t_ value of the GAPDH control from the same RNA preparation. The ratio of transcription of each gene was calculated as 2^‐(ΔCt)^, where ΔC_t_ is the difference C_t(test gene)_−C_t(GAPDH)_. Real-time RT-PCR primer sequences used in this study were: N-cadherin F-5’-ACAGTGGCCACCTACAAAGG-3’, R-5’-CCGAGATGGGGTTGATAATG-3’, E-cadherin F-5’-ACGTCGTAATCACCACACTGA-3’, R-5’-TTCGTCACTGCTACGTGTAGAA-3’, GAPDH F-5’-CCATGGAGAAGGCTGGGG -3’, R-5’-CAAAGTTGTCATGGATGACC -3’.

**Animal studies**

Ethics Statement: The animal use protocol listed below has been reviewed and approved by Institutional Animal Care and Use Committee (IACUC), Hualien Tzu Chi Hospital, approval No: 104-13. To examine the anti-tumor effects of BP *in vivo*, the BFTC human bladder cancer cells were used in male NOD-SCID mice experiments. The mice were bred and maintained in the Laboratory Animal Center at Tzu Chi University (Hualien, Taiwan). All procedures were performed in compliance with the standard operating procedures of the Tzu Chi University Laboratory Animal Center (Hualien, Taiwan). All experiments were carried out using 6–8 week old mice weighing 18–22 g. The animals were subcutaneous implanted with 5 x 10^5^ BFTC cells into the back of mice. When the tumor reached 100–150 mm^3^ in volume, animals were divided randomly into control and test groups consisting of six mice per group (day 0). Subcutaneous injection of either corn oil (control group), or BP (100 and 200 mg/kg, treatment groups) was given for five successive days. The injection sites were > 1.5 cm from the tumors. Mice were weighed three times a week up to day 31. The tumor volume was also determined by measurement of the length (L) and width (W) of the tumor. The tumor volume at day n (TVn) was calculated as TV (mm^3^) = (L x W^2^)/2. The relative tumor volume at day n (RTVn) versus day 0 was expressed according to the following formula: RTVn= TVn/TV0. Tumor regression (T/C (%)) in treated versus control mice was calculated using: T/C (%) = (mean RTV of treated group)/(mean RTV of control group) x 100. Xenograft tumors as well as other vital organs of treated and control mice were harvested and fixed in 4% formalin, embedded in paraffin, and cut in 4-mm sections for histological analysis.
